# Supplementary material for: Impaired cellular energy metabolism in cord blood macrophages contributes to abortive response toward inflammatory threats
Source: Nat Commun. 2019 Apr 11;10:1685. doi: 10.1038/s41467-019-09359-8 (PMC6459909; doi:10.1038/s41467-019-09359-8)
Supplement: Supplementary file 2 — Supplementary Information [file 41467_2019_9359_MOESM2_ESM.pdf]

Supplementary Information:

Impaired cellular energy metabolism in neonatal macrophages contributes to abortive response toward inflammatory threats

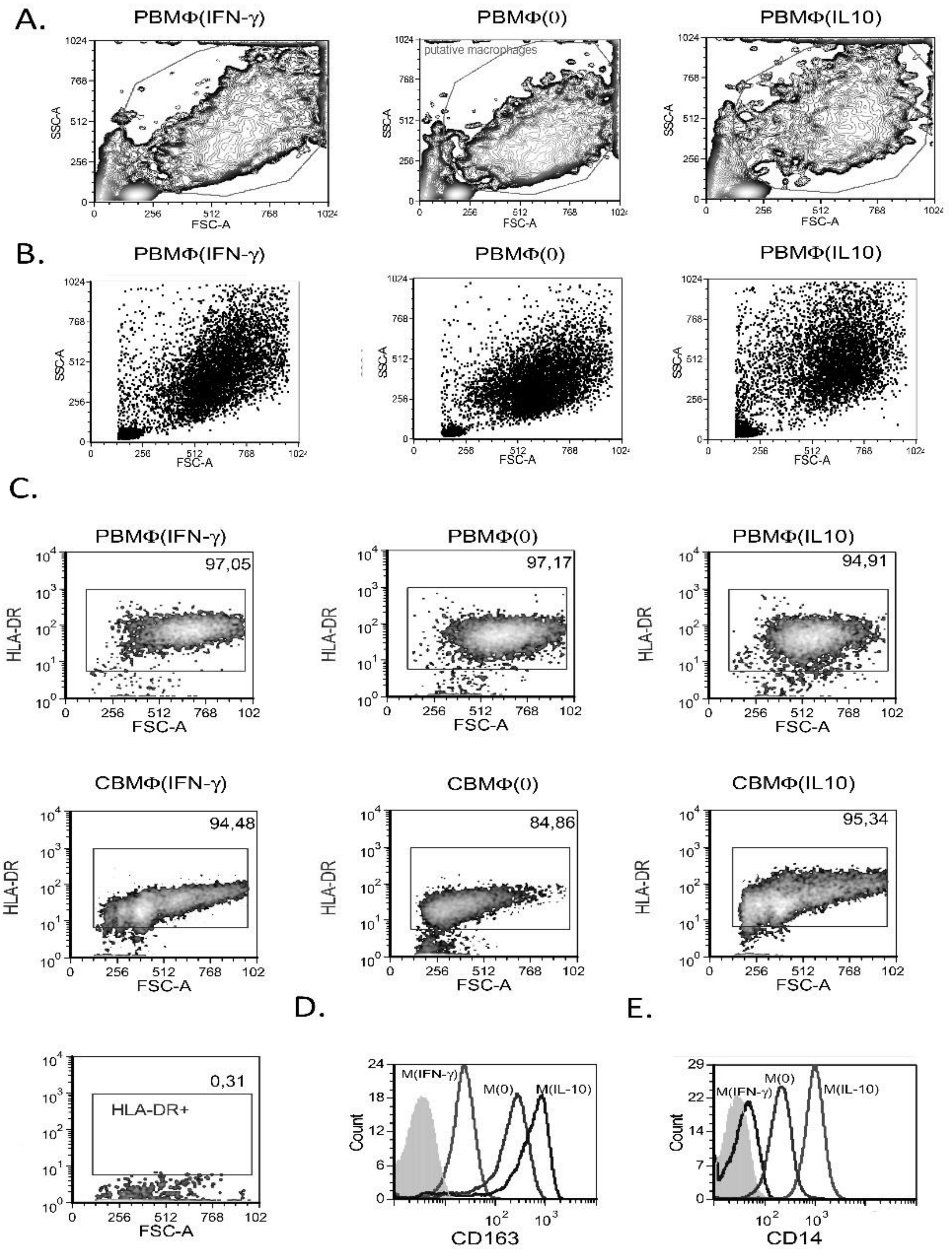
A.
PBMΦ(IFN-γ)
PBMΦ(0)
PBMΦ(IL10)
putative macrophages
SSC-A
FSC-A
B.
PBMΦ(IFN-γ)
PBMΦ(0)
PBMΦ(IL10)
C.
PBMΦ(IFN-γ)
97,05
PBMΦ(0)
97,17
PBMΦ(IL10)
94,91
CBMΦ(IFN-γ)
94,48
CBMΦ(0)
84,86
CBMΦ(IL10)
95,34
HLA-DR
FSC-A
D.
E.
0,31
HLA-DR+
M(IFN-γ)
M(0)
M(IL-10)
Count
CD163
CD14

F

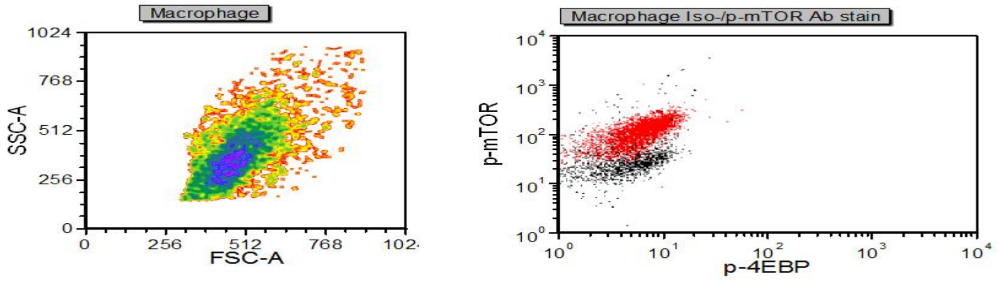

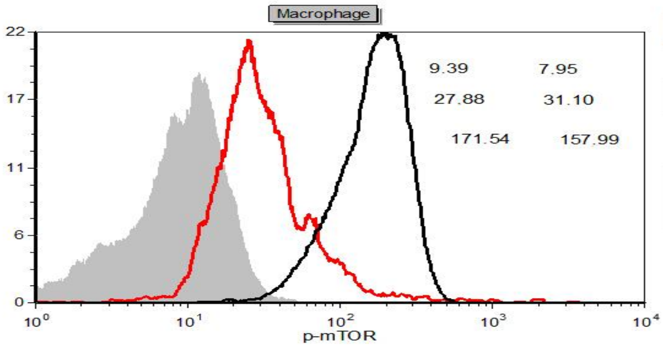

	Median	Mean
no staining	93.9	79,5
Isotype-Ab	278,8	311.0
p-mTOR	1715.4	1579.9
Δ Isotype-Ab/ p-mTOR	1436.6	1268.9

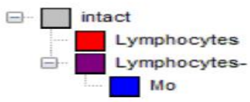

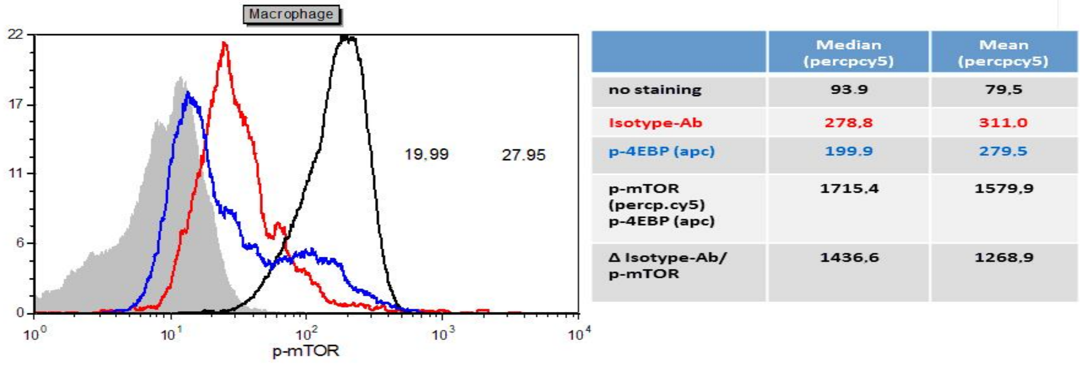

	Median (percpcy5)	Mean (percpcy5)
no staining	93.9	79,5
Isotype-Ab	278,8	311.0
p-4EBP (apc)	199.9	279.5
p-mTOR (percp.cy5) p-4EBP (apc)	1715.4	1579.9
Δ Isotype-Ab/ p-mTOR	1436.6	1268.9

G

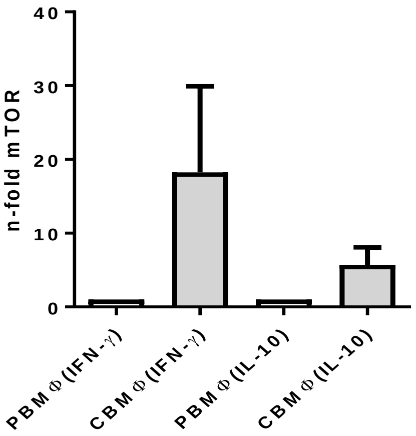

Supplementary Figure 1: Gating strategy and isotype control staining used to determine protein expression by flow cytometry and mTOR mRNA expression. A-B) Forward- sideward scatter analysis of putative PBMΦ preparations. Cytokines used for polarization are indicated above the density plots (A) and the dot plots, in which cellular debris was subtracted (B). HLA-DR expressing cells were designated as putative macrophages (compare gate in (A)). Representative percentage of HLA-DR positive cells are given for PBMΦ and CBMΦ (C). HLA-DR positive cells were analyzed for CD163 and CD14 expression (histograms in D and E, polarization of PBMΦ as indicated), respectively. Representative staining of pMTOR and p 4EBP-1 is shown in (F). G) N-fold mRNA expression of *mTOR* in CBMΦ(IFN-γ) (n=3) and CBMΦ(IL-10)Φ (n=3) compared to means of PBMΦ(IFN-γ) (n=4) and PBMΦ(IL-10) (n=5) analyzed by RT-qPCR. Bars indicate mean and error bars SD.

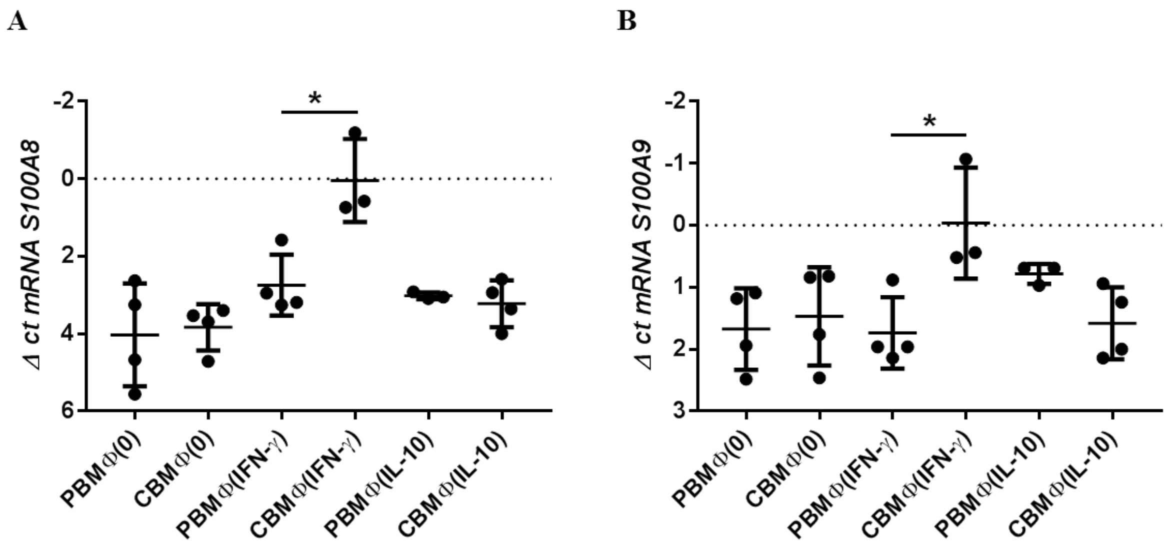

Supplementary Figure 2: S100A8/9 mRNA expression in CBMΦ compared to PBMΦ. A) Δ ct level of *S100A8* mRNA expression in n=4/n=3 independent samples, mRNA expression was determined after normalization to the endogenous reference control gene ß-actin. Bars indicate mean and error bars SD, two-tailed, unpaired t-test, *p=0,016. B) Δ ct level of *S100A9* mRNA expression in n=4/n=3 independent samples, mRNA expression was determined after normalization to the endogenous reference control gene ß-actin. Bars indicate mean and error bars SD, two-tailed, unpaired t-test, *p=0,0236.

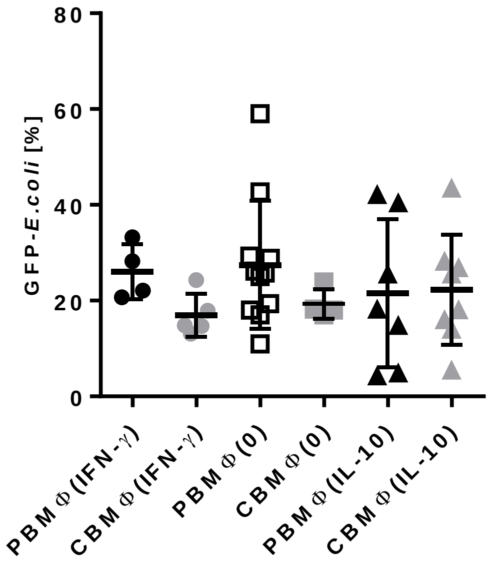

Supplementary Figure 3: Phagocytic indices of indicated groups incubated with GFP-E.coli at an multiplicity of infection (MOI) of 25 for 1h. For PBMΦ independent N was 4, 11, 7 and 3, for CBMΦ 5, 4, 8 and 3, respectively.

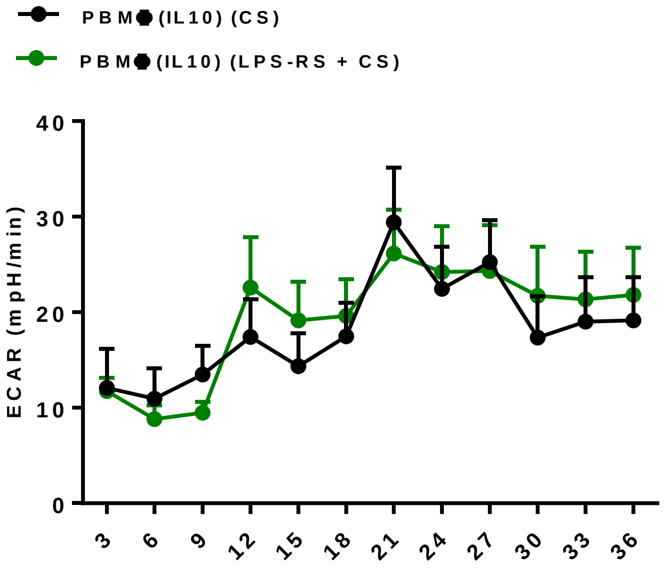

Supplementary Figure 4: Treatment with LPS-RS cannot abrogate ECAR levels in MΦ(IL-10) after incubation with CS. ECAR levels were measured after incubation of MΦ(IL10) Φ with CB serum (CS) +/- LPS-RS, Points indicate mean from 3 experiments, error bars SEM.

Supplementary Figures 5 - 9

A

	CD14	TLR4	HLA-DR	CD80	CD86	IFNy-R
N = individual PBMΦ(0)	4	3	6	4	3	4
N = individual CBMΦ(0)	5	4	4	3	4	3
P value	0.0002	0.003	n.s.	0.0001	n.s.	n.s.

B

	CD16	CD32	CD64	CD163	CD206	IL10-R	Arg	p-STAT3
N = individual PBMΦ(0)	5	5	3	4	3	3	4	4
N = individual CBMΦ(0)	4	3	6	8	3	3	4	6
P value	0.022	0.04	0.001	0.001	n.s.	n.s	0.041	n.s.

C

	CD14	TLR4	HLA-DR	CD80	CD86	IFNy-R
N = individual PBMΦ(IFNy)	4	3	4	5	4	3
N = individual CBMΦ(IFNy)	3	3	4	4	3	3
P value	0.046	n.s.	0.0007	0.0002	0.0001	n.s.

D

	CD16	CD32	CD64	CD163	CD206	IL10-R	Arg	p-STAT3
N = individual PBMΦ(IL10)	4	3	4	8	3	4	6	5
N = individual CBMΦ(IL10)	3	3	5	3	3	3	3	3
P value	0.22	0.04	0.001	0.001	n.s.	n.s	0.041	n.s.

Supplementary Figure 5: Tables give numbers of individual samples of the indicated surface and intracellular markers shown in Figure 1. P-values of one-way ANOVA tests are given in the bottom line.

A

time	3	6	9	12	15	18	21	24	27	30	33	36
N = individual PBMΦ(IFNy)	3	3	3	3	3	3	3	3	3	3	3	3
N = individual CBMΦ(IFNy)	4	4	3	4	4	4	4	4	4	4	4	4
P-values	n.s.	n.s.	0.002	0.003	0.008	0.0012	0.0001	0.0002	0.0002	0.0009	0.0036	0.0056

B

time	3	6	9	12	15	18	21	24	27	30	33	36
N = individual PBMΦ(IL-10)	3	3	3	3	3	3	3	3	3	3	3	3
N = individual CBMΦ(IL-10)	4	4	4	4	4	4	4	4	4	3	3	3
P-values	0.091	0.0001	0.0001	0.0001	0.0001	0.0001	0.0001	0.0001	0.0001	0.0001	0.0001	0.024

C

time	3	6	9	12	15	18	21	24	27	30	33	36
N = individual PBMΦ(IFNy)	3	3	3	3	3	3	3	3	3	3	3	3
N = individual CBMΦ(IFNy)	3	3	3	3	3	3	3	3	3	3	3	4
P-values	0.001	0.001	0.001	n.s.	n.s.	n.s.	n.s.	0.0001	0.001	0.0001	0.0001	0.002

D

time	3	6	9	12	15	18	21	24	27	30	33	36
N = individual PBMΦ(IL-10)	3	3	3	3	3	3	3	3	3	3	3	3
N = individual CBMΦ(IL-10)	3	3	3	3	3	3	3	3	3	3	3	3
P-values	n.s.	n.s.	n.s.	n.s.	n.s.	n.s.	n.s.	n.s.	n.s.	n.s.	n.s.	n.s.

Supplementary Figure 6: Tables give numbers of individual heptaplicates of ECAR (upper tables A-B) and OCR (lower tables C-D) as shown in Figure 3. P-values of one-way ANOVA tests are given in the bottom line.

	PBMΦ(IFN-y)	CBMΦ(IFN-y)	PBMΦ(0)	CBMΦ(0)	PBMΦ(IL-10)	CBMΦ(IL-10)
**N**	**3**	**3**	**4**	**6**	**4**	**4**
**P-value**	0.042		0.0004		0.0006	

	PBMΦ(IFN-y)	CBMΦ(IFN-y)	PBMΦ(0)	CBMΦ(0)	PBMΦ(IL-10)	CBMΦ(IL-10)
**N**	**4**	**5**	**3**	**3**	**3**	**3**
**P-value**	0.04		0.004		0.0006	

	PBMΦ(IFN-y)	CBMΦ(IFN-y)	PBMΦ(0)	CBMΦ(0)	PBMΦ(IL-10)	CBMΦ(IL-10)
**N**	**5**	**3**	**7**	**5**	**10**	**4**
**P-value**	n.s.		n.s.		0.0046	

	PBMΦ(IFN-y)	CBMΦ(IFN-y)	PBMΦ(0)	CBMΦ(0)	PBMΦ(IL-10)	CBMΦ(IL-10)
**N**	**4**	**3**	**8**	**4**	**4**	**6**
**P-value**	0.03		0.002		0.0027	

Supplementary Figure 7: Tables give numbers of individual samples used for determining mTOR (upper table), p-mTOR (second table), p-S6 (third table) and p4EBP (lowest tabel) as presented in Figure 4. P-values of one-way ANOVA tests are given in the bottom line.

E

time	3	6	9	12	15	18	21	24	27	30	33	36
N = individual PBMΦ (IFN-y)	6	6	6	5	6	6	4	4	4	5	5	5
N = individual PBMΦ (IFN-y) + Rapamycin	3	3	3	3	3	3	3	3	3	3	3	3
P-values (student`s t-test)	n.s.	n.s.	n.s.	n.s.	n.s.	n.s.	0.0091	n.s.	n.s.	n.s.	n.s.	n.s.

F

time	3	6	9	12	15	18	21	24	27	30	33	36
N = individual PBMΦ (IL-10)	3	3	3	3	3	3	3	3	3	3	3	3
N = individual PBMΦ (IL-10) + Rapamycin	4	3	4	4	4	4	4	4	4	4	4	4
P-values (student`s t-test)	n.s.	n.s.	n.s.	n.s.	n.s.	n.s.	n.s.	n.s.	n.s.	n.s.	n.s.	n.s.

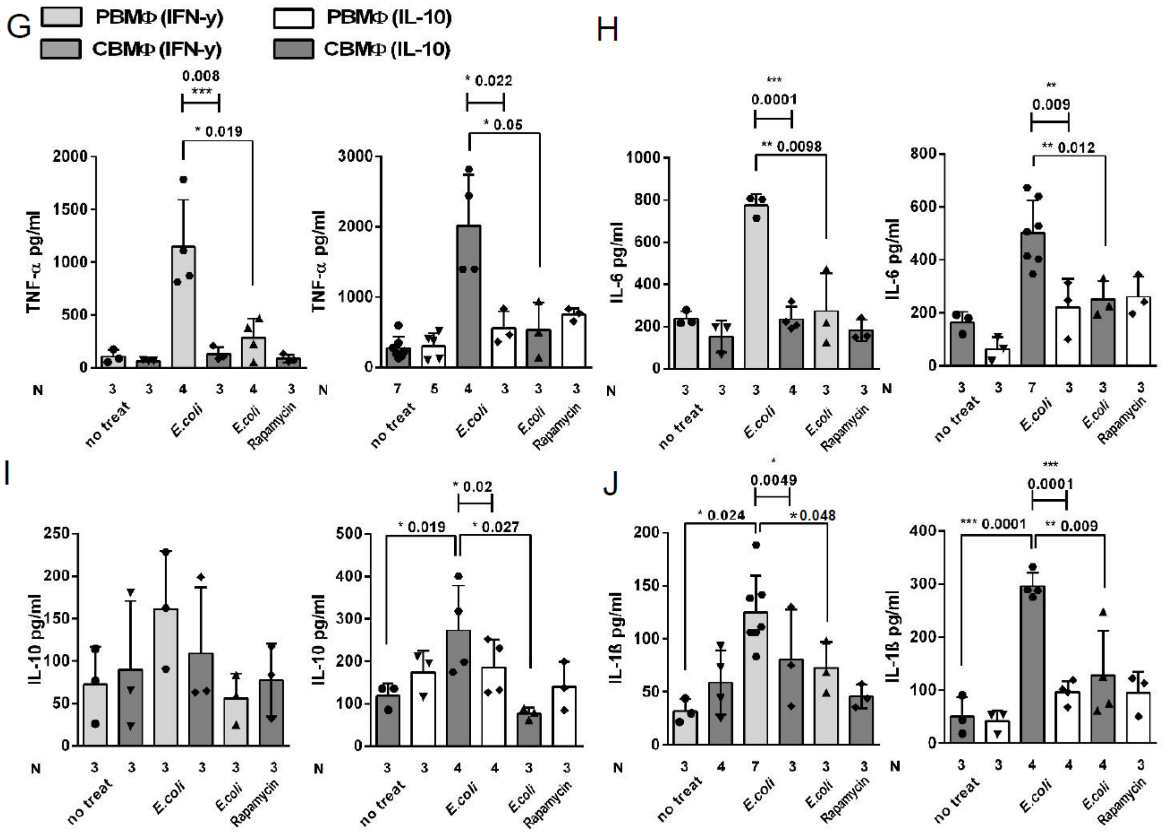

Supplementary Figure 8: Tables give numbers of individual heptaplicates of ECAR assays from indicated MΦ (addition to Figure 5, E-F). P-values of one-way ANOVA tests are given in the bottom line. ELISA assay as shown in Figure 5 (G-J) with additional individual sample numbers given below the chart. Individual p-values of student`s t-tests and one-way ANOVA tests (blunt ended bars) are added.

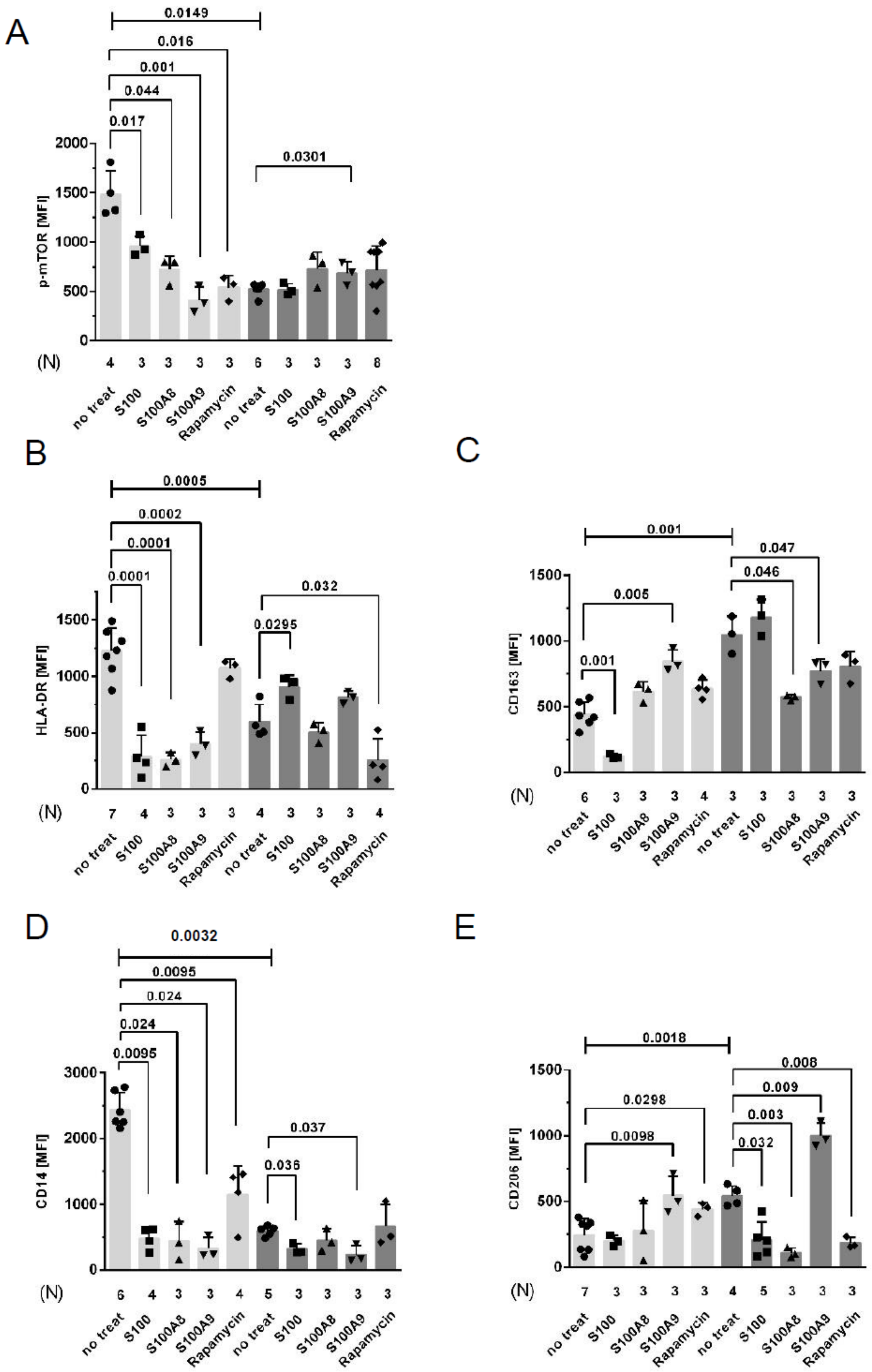
A
B
C
D
E
p-mTOR [MFI]
HLA-DR [MFI]
CD163 [MFI]
CD14 [MFI]
CD206 [MFI]
(N)
no treat
S100
S100A8
S100A9
Rapamycin
0.0149
0.016
0.001
0.044
0.017
0.0301
0.0005
0.0002
0.0001
0.0001
0.032
0.0295
0.001
0.047
0.046
0.005
0.001
0.0032
0.0095
0.024
0.024
0.0095
0.037
0.036
0.0018
0.008
0.009
0.0298
0.003
0.0098
0.032

F

time	3	6	9	12	15	18	21	24	27	30	33	36
N = individual PBMΦ(IFN-y)	6	6	6	5	6	6	3	3	3	6	6	6
N = individual PBMΦ(IFN-y+S100)	4	4	4	4	4	4	4	4	4	3	3	3
P-values (student`s t-test)	n.s.	n.s.	n.s.	0.039	n.s.	n.s.	0.019	0.005	0.013	n.s.	n.s.	n.s.

G

time	3	6	9	12	15	18	21	24	27	30	33	36
N = individual PBMΦ(IL-10)	5	4	5	4	5	5	4	4	4	5	5	5
N = individual PBMΦ(IL-10) + S100	3	4	4	4	4	4	4	4	4	4	4	4
P-values (student`s t-test)	n.s.	0.027	0.001	0.0001	0.0001	0.0001	0.0001	0.0001	0.0001	0.0001	0.024	n.s.

H

time	3	6	9	12	15	18	21	24	27	30	33	36
N = individual PBMΦ (IFN-y + CS)	3	3	3	3	3	3	3	3	3	3	3	3
N = individual PBMΦ (IFN-y + LPS-RS + CS)	3	3	3	3	3	3	3	3	3	3	3	3
P-values (student`s t-test)	n.s.	n.s.	n.s.	n.s.	n.s.	n.s.	n.s.	n.s.	n.s.	n.s.	n.s.	n.s.

Supplementary Figure 9:

Flow cytometric assay as shown in Figure 6 (A-E) with additional individual sample numbers given below the chart. Individual p-values of student`s t-tests and one-way ANOVA tests (blunt ended bars) are added. Tables give numbers of individual heptaplicates of ECAR assays from indicated MΦ (Figure 6 F-H). P-values are given in the bottom line.

Supplementary Table 1: Enriched KEGG pathways in CBMΦ(IL-10) and Rapamycin treated MΦ(IL-10) compared to adult macrophages (p<0,05, R<2). Pathways that are regulated in both groups are highlighted

CBMΦ(IL-10)	N-fold	Rapamycin treated MΦ(IL-10)	N-fold
**Steroid biosynthesis**	6,55	**Steroid biosynthesis**	8,56
**Propanoate metabolism**	6,38	Terpenoid backbone biosynthesis	7,78
**Lysine degradation**	5,76	DNA replication	7,73
**Terpenoid backbone biosynthesis**	4,71	Fatty acid biosynthesis	6,59
**Pyruvate metabolism**	4,03	Systemic lupus erythematosus	5,07
**Fatty acid metabolism**	3,95	Cell cycle	4,83
**Fatty acid elongation**	3,95	**Antifolate resistance**	4,14
**Protein export**	3,95	Alcoholism	3,92
**One carbon pool by folate**	3,89	Proteasome	3,89
**Valine, leucine and isoleucine degradation**	3,69	**Pyruvate metabolism**	3,84
**Dorso-ventral axis formation**	3,07	Homologous recombination	3,66
**Notch signaling pathway**	2,96	**Fatty acid metabolism**	3,57
**Fatty acid degradation**	2,89	Fanconi anemia pathway	3,50
**beta-Alanine metabolism**	2,86	Toll-like receptor signaling pathway	3,23
**Protein processing in endoplasmic reticulum**	2,84	p53 signaling pathway	3,10
**Antifolate resistance**	2,76	Viral carcinogenesis	2,51
**Porphyrin and chlorophyll metabolism**	2,68	Chemokine signaling pathway	2,18
**Tryptophan metabolism**	2,30	Cytokine-cytokine receptor interaction	2,10
**Carbon metabolism**	2,26		
**Parkinson's disease**	2,06		

Supplementary Table 2: Enriched KEGG pathways in CBMΦ(IL-10) and Rapamycin treated MΦ(IL-10) compared to adult macrophages ($p<0,05$, $R<2$).

CBMΦ (IFN-γ)	N-fold	Rapamycin treated MΦ(IFN-γ)	N-fold
**Asthma**	6,10	Steroid biosynthesis	12,35
**Allograft rejection**	4,81	Terpenoid backbone biosynthesis	8,42
**Graft-versus-host disease**	4,81	Pyruvate metabolism	4,75
**Type I diabetes mellitus**	4,17	Fatty acid metabolism	4,50
**Antigen processing and presentation**	3,90	TNF signaling pathway	4,49
**Vasopressin-regulated water reabsorption**	3,60	Inflammatory bowel disease (IBD)	4,28
**Prion diseases**	3,60	Lysosome	3,77
**Hematopoietic cell lineage**	3,30	Chagas disease (American trypanosomiasis)	3,27
**Autoimmune thyroid disease**	3,30	Osteoclast differentiation	2,80
**Amphetamine addiction**	3,15	Cytokine-cytokine receptor interaction	2,33
**Viral myocarditis**	3,05		
**Estrogen signaling pathway**	2,61		
**Th17 cell differentiation**	2,43		
**Dopaminergic synapse**	2,41		
**Natural killer cell mediated cytotoxicity**	2,33		
**Herpes simplex infection**	2,16		
**Endocytosis**	2,15		
**Cytokine-cytokine receptor interaction**	2,10		
**Tuberculosis**	2,03		
